# Tissierella Bacteremia in the Setting of Polymicrobial Osteomyelitis

**DOI:** 10.7759/cureus.74230

**Published:** 2024-11-22

**Authors:** Emma M Griffith, Hamel Patel, Vandana Seeram

**Affiliations:** 1 Internal Medicine, University of Florida College of Medicine – Jacksonville, Jacksonville, USA; 2 Pulmonary and Critical Care Medicine, University of Florida College of Medicine – Jacksonville, Jacksonville, USA

**Keywords:** infectious disease medicine, osteomyelitis, polymicrobial osteomyelitis, septic shock [ss], tissierella praeacuta

## Abstract

We present a case of *Tissierella* bacteremia in the setting of polymicrobial osteomyelitis. *Tissierella* is a Gram-variable bacterium that has been rarely documented as the etiologic organism in human infections such as septic arthritis or otitis media, and even more rarely reported as an organism associated with bacteremia. The patient presented with septic shock and the physical exam was notable for gangrene of bilateral feet. Infectious workup was significant for acute osteomyelitis and septic arthritis of his feet in the setting of *Tissierella *bacteremia*, *which was successfully treated with piperacillin-tazobactam and then metronidazole in addition to surgical resection of the feet.

## Introduction

*Tissierella *spp.* *is a Gram-variable anaerobic bacterium that has been rarely documented as the etiologic organism in human infections with less than 10 reported cases [1]. With recent advances in technology such as matrix-assisted laser desorption/ionization-time of flight mass spectrometry (MALDI-TOF), more bacterial species are being reported by microbiology departments [2]. *Tissierella *spp. has been reported as the source of bacteremia in two other cases per the literature review [1,3]. It is a member of the *Clostridiales* genus, and was originally isolated in 1908 and classified as *Clostridium *species. [1,4]. Only the species *Tissierella praeacuta *has been shown to cause pathology in humans, and it can be either Gram-positive or Gram-negative.* Tissierella creatinini* and *Tissierella creatinophila *are other *Tissierella *species that have been isolated, but they have not been demonstrated as etiologic organisms of infections in humans [5,6]. Documented infections due to *Tissierella praeacuta *in humans* *include sacral wound infection, septic arthritis, liver abscess, otitis media, and pyometra. Known sources of *Tissierella praeacuta *include soil and the human gastrointestinal tract. The following antibiotics have been documented as successful treatment options for *Tissierella *infection: piperacillin-tazobactam, metronidazole, chloramphenicol, meropenem, and rifampin [3,7].

## Case presentation

**Figure 1:**
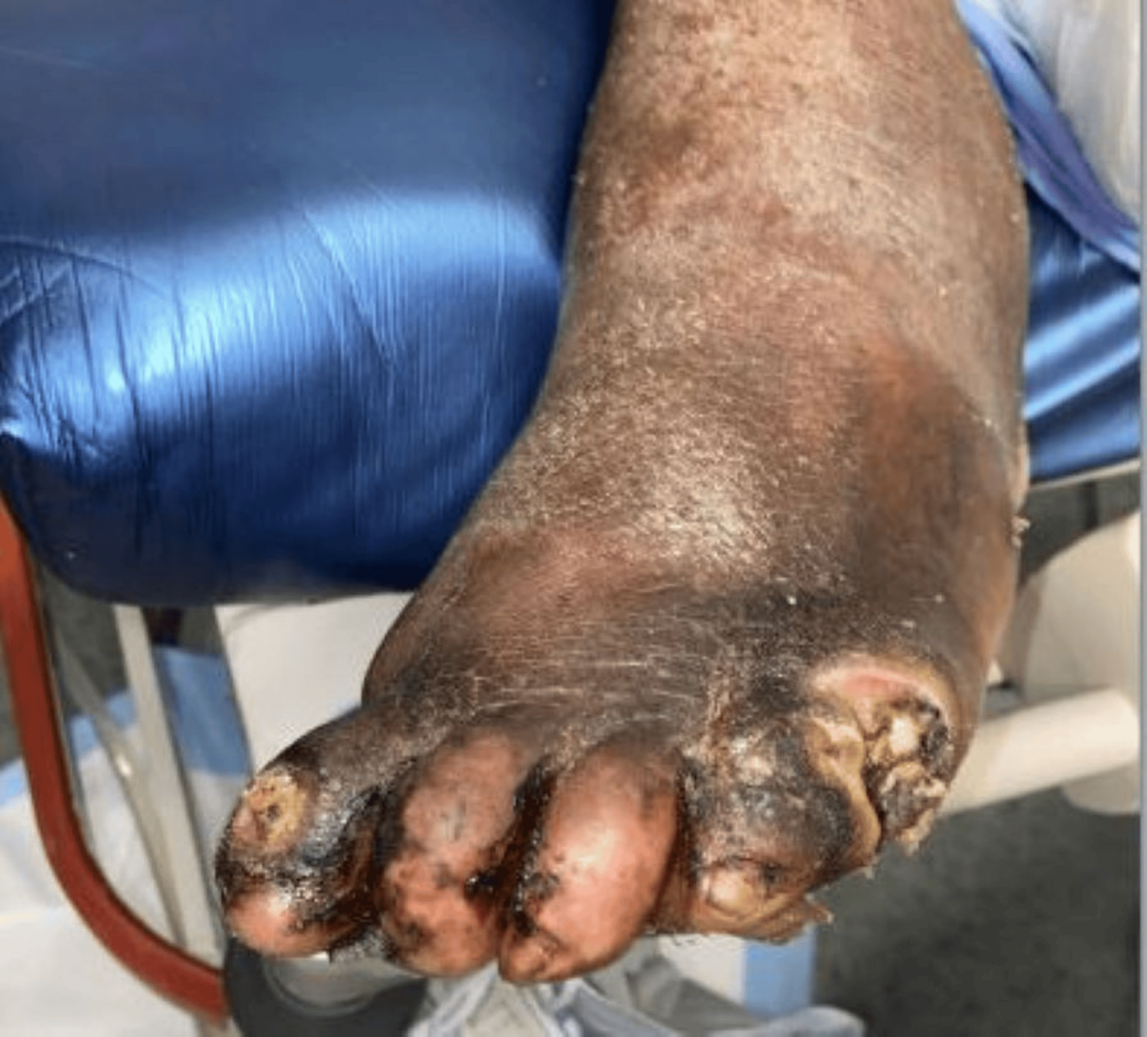
Appearance of left foot on examination

A 68-year-old male patient with a past medical history of left great toe amputation due to osteomyelitis, substance use disorder, and alcohol use disorder presented to the emergency department with altered mental status after alcohol consumption. On initial exam, he was somnolent and hypothermic to 85˚F with gangrene of bilateral feet complicated by exposed bone, which is shown in Figure 1.

Laboratory studies were significant for leukocytosis, anion gap metabolic acidosis due to lactic acidosis, and elevated C-reactive protein, which are listed in Table 1. Urinalysis and chest X-ray were negative for signs of infection. His hemoglobin A1c was within normal limits. 

**Table 1:** Laboratory results

Laboratory test	Result	Reference range
White blood cell count (cells/mm^3^)	32,950	4,500-11,000
Complete blood count differential (neutrophils)	91%	34-73%
Lactic Acid (mmol/L)	9.7	0.7-2.7
C-reactive protein (mg/L)	91	0.1-2.8

**Figure 2:**
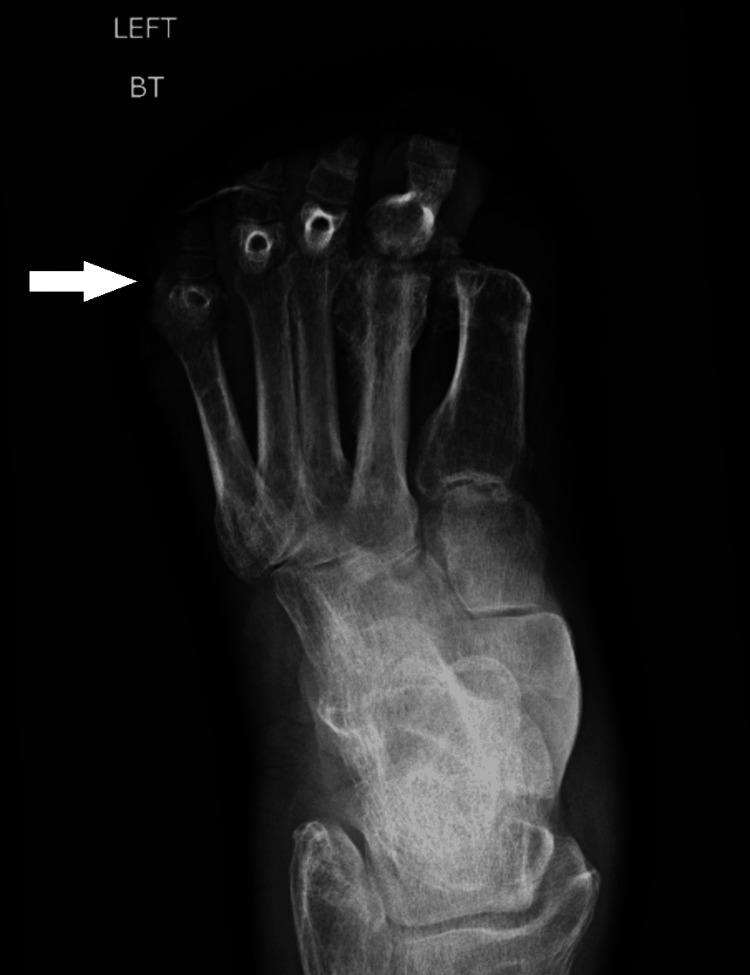
X-ray of the left foot Anterior-posterior (AP) view of the left foot shows prior amputation of the first distal phalanx in addition to cortical irregularity at the base of the fifth proximal phalanx (marked by the arrow).

The X-ray of bilateral feet showed cortical irregularity of the left fifth proximal phalanx concerning for osteomyelitis as shown in Figure 2. Computed tomography with angiography (CTA) of the bilateral lower extremities was negative for arterial flow limitation, but showed acute osteomyelitis with subcutaneous emphysema in his right great toe and possible osteomyelitis of left foot. The patient was started on empiric treatment for sepsis with vancomycin and piperacillin-tazobactam and then admitted to intermediate care. His mentation improved with intravenous fluids and correction of hypothermia. Magnetic resonance imaging (MRI) of the bilateral feet was performed and showed septic arthritis of the left fifth metatarsophalangeal joint with adjacent cellulitis and osteomyelitis. MRI also confirmed early osteomyelitis of the right foot. 

He subsequently developed hypotension despite fluid resuscitation. So he was transferred to the intensive care unit for further management of septic shock complicated by metabolic acidosis. The initial blood cultures grew anaerobic Gram-positive rods suggestive of diphtheroids, which were identified as *Tissierella spp*. via MALDI-TOF mass spectrometry. Susceptibilities of *Tissierella* were not reported. On the day nine of hospitalization, he underwent surgical resection of the bilateral feet including right first partial ray and left second and fifth partial ray resections then subsequent right trans-metatarsal amputation. The pathology showed acute osteomyelitis with viable margins. The tissue cultures were polymicrobial including methicillin-resistant *Staphylococcus aureus, Corynebacterium *spp., and coagulase-negative *Staphylococcus*. Repeat blood cultures on day five of hospitalization showed no growth after treatment with vancomycin and piperacillin-tazobactam. After 12 days of intravenous antibiotics, he was switched from intravenous vancomycin and piperacillin-tazobactam to oral metronidazole in order to de-escalate his regimen after negative blood cultures. Later in admission, he was taken for definitive surgical treatment with right trans-metatarsal amputation and left fifth ray disarticulation. He remained afebrile and hemodynamically stable. His antimicrobial regimen was changed at hospital discharge to minocycline and metronidazole for a total of six weeks for treatment of polymicrobial osteomyelitis of bilateral feet and septic arthritis of left foot. 

## Discussion

This patient’s initial presentation was non-specific with regards to sepsis, which progressed to septic shock due to lack of infectious source control. He had no history of immunocompromising conditions or infection due to resistant organisms, but he did have a previous episode of osteomyelitis requiring amputation which increased his risk for bacteremia [8]. Intraoperative cultures collected from his feet did not grow *Tissierella*, which is likely due to preoperative antibiotic exposure. The lack of identification via culture does not rule out osteomyelitis as the source of his bacteremia, and additional imaging including chest X-ray and CT did not elucidate another etiology.

His *Tissierella *infection was successfully treated with a course of piperacillin-tazobactam as well as surgical resection of the infected tissues in his feet due to underlying septic arthritis and osteomyelitis. As per the literature review, this is consistent with other cases of *Tissierella*, specifically its susceptibility to beta-lactams and metronidazole [9]. Table 2 lists the antibiotics utilized in other case reports to successfully treat infections with *Tissierella praeacuta*. As with other Gram-negative bacteremia, this infection cleared with one week of appropriate intravenous antimicrobial therapy [10]. Due to the presence of acute osteomyelitis and septic arthritis in this case, antimicrobial treatment was prolonged for a total duration of six weeks from the date of surgical intervention (day nine of hospitalization) for infectious source control. The initial part of the antibiotic course was twelve days of IV piperacillin-tazobactam (in addition to IV vancomycin for empiric coverage) before transitioning to oral metronidazole. His hospital course was prolonged and he was started on minocycline three weeks after his surgery in preparation for hospital discharge to cover methicillin-resistant *Staphylococcus aureus*.

**Table 2:** Summary of antimicrobial therapy administered for Tissierella praeacuta in case reports

Author	Infectious source	Antibiotics used
Gill et al. [1]	Bacteremia and osteomyelitis	Metronidazole, levofloxacin, cefepime, amoxicillin-clavulanate
Yang et al. [3]	Sacral wounds	Piperacillin-tazobactam, levofloxacin, metronidazole
Camelena et al. [7]	Prosthetic knee joint infection	Piperacillin-tazobactam, metronidazole
Camelena et al. [7]	Bacteremia and liver abscess	Piperacillin-tazobactam, meropenem
Ørum et al. [9]	Bacteremia and pyometra	Piperacillin-tazobactam

This bacteria would likely have been misclassified as *Clostridium *spp. without the use of MALDI-TOF mass spectrometry, as the Gram stain and culture did not identify *Tissierella *spp. specifically. An alternative method for identifying bacteria is 16S ribosomal RNA sequencing, which can be useful if MALDI-TOF mass spectrometry or traditional culture methods fail to identify a bacterium within a mixed sample [11]. With the increasing use of MALDI-TOF mass spectrometry, there will likely be an increasing amount of *Tissierella *infections identified in the future. Further research should focus on specific antibiotics as well as their susceptibilities for treatment of this organism in order to elucidate an effective treatment regimen.

## Conclusions

*Tissierella *is a rarely reported source of bacteremia which can lead to serious complications, such as septic shock in this case. It can be identified via MALDI-TOF mass spectrometry after growth on cultures. Treatment of *Tissierella *should involve surgical resection of infected tissue for source control in addition to intravenous antibiotics such as metronidazole, meropenem, or piperacillin-tazobactam. This noteworthy case of *Tissierella *bacteremia was successfully treated with piperacillin-tazobactam. At this time, literature on this pathogen consists solely of case reports. Future investigation should focus on establishing the treatment of choice for this microorganism in order to delineate guidelines for management.
